# When Nerves Tell a Different Story: Functional Neurological Disorder (FND) After a Minor Hand Injury

**DOI:** 10.7759/cureus.61218

**Published:** 2024-05-28

**Authors:** Ioannis Kyriazidis, Efterpi Demiri, Charikleia Mitsaki, Dimitrios Dionyssiou

**Affiliations:** 1 Department of Plastic and Reconstructive Surgery, General Hospital Papageorgiou, Thessaloniki, GRC; 2 Department of Plastic and Reconstructive Surgery, School of Medicine, Faculty of Health Sciences, Aristotle University of Thessaloniki, Thessaloniki, GRC

**Keywords:** loss of hand function, transient hand dysfunction, hand trauma, functional movement disorder, functional neurological disorder

## Abstract

Hand injuries typically present with localized symptoms. However, we report an unusual case of a 32-year-old female who experienced a transient complete loss of sensation and motor function in her entire left nondominant hand after sustaining a minor 1 cm stab wound between the third and fourth metacarpals.

Wound exploration under local anesthesia revealed no tendon, vascular, neural, or bony injury. Remarkably, she spontaneously regained full hand sensation and function within 120 minutes of the injury.

Extensive neurological evaluation, including magnetic resonance imaging (MRI), electromyography (EMG), nerve conduction studies (NCS), and somatosensory evoked potentials (SSEPs), ruled out organic pathology and supported a diagnosis of functional neurological disorder (FND), specifically functional movement disorder (FMD).

Close collaboration between hand surgeons, neurologists, and occupational therapists is essential for accurate diagnosis and appropriate multidisciplinary management.

Further research is needed to elucidate the mechanisms underlying FND and optimize evidence-based treatment for FND in the context of hand trauma. The increased awareness of this condition across specialties involved in hand injury management is crucial to facilitate timely diagnosis and avoid unnecessary interventions.

## Introduction

Hand injuries primarily manifest with localized symptoms [1-3]. However, unique cases can unveil the sophisticated neurological and physiological interactions during the body's response to injury. We discuss a unique case of transient and extensive numbness and active range of motion (aROM) loss following minor hand trauma, indicative of functional neurological disorder (FND), a disorder at the interface between neurology and psychiatry​ [4].

## Case presentation

A 32-year-old right-hand-dominant, nonsmoking female, a mother of two with no significant past medical or psychiatric history and no prior hand injuries or surgeries, arrived at the emergency department 60 minutes post injury to her left hand. The isolated stab injury between the third and fourth metacarpals in flexor zone 3 on her left palm resulted in a 1 cm wound without tissue loss, ongoing bleeding, or hematoma. Upon initial examination, the patient exhibited complete loss of sensation and aROM of the entire left hand, including the thumb and all long fingers. The patient's initial presentation and subsequent recovery are demonstrated in Video 1. The initial neurological examination revealed the following: Reflexes show normal biceps, triceps, and brachioradialis reflexes bilaterally; muscle strength shows 0/5 in all muscles of the left hand, 5/5 in the left wrist and arm muscles, and 5/5 in all right upper extremity muscles ("Loss of aROM" section of Video 1); and sensory testing shows complete loss of light touch and pinprick in all dermatomes of the left hand and normal sensation in the left forearm and right upper extremity ("Loss of Sensation" section of Video 1).

**Video 1:** Patient's initial presentation and clinical findings upon initial presentation and after the gradual recovery within 120 minutes post injury. A 32-year-old female presented with transient hand dysfunction following a minor stab injury. The video demonstrates the initial complete loss of sensation and active range of motion, followed by the gradual recovery of function within 120 minutes post injury.

Notably, the examination did not reveal any abnormal temperature of the left hand, swelling, vascular compromise, nor intrinsic muscle spasms. Following the examination, she was referred for X-ray evaluation, which revealed no pathological findings or presence of a foreign body. The patient's history was free from medical or psychiatric comorbidities, and the patient had no history of allergies and was not on any medication.

The treatment was concluded with an exploration of the wound under local anesthesia, revealing no tendon, vascular, neural, or bone injury. Approximately 90 minutes post injury, the patient gradually began regaining sensation and movement in the left hand movement ("Recovery" section of Video 1). By 120 minutes post injury, the patient had fully recovered sensation and motor function in the affected hand. A follow-up examination at 48 hours post injury revealed no residual sensory or motor deficits.

Neurological evaluation and diagnosis confirmation

Upon suspicion of functional neurological disorder (FND), the patient was referred to a neurologist for a comprehensive evaluation. The neurologist conducted a thorough clinical assessment, including a detailed history taking and a focused neurological examination. The patient's medical history was reviewed, and no prior neurological or psychiatric conditions were identified. The patient did not report any similar symptomatology in the past.

The neurologist performed a structured neurological examination and diagnostic tests to rule out other potential neurological conditions. These tests are shown below.

Magnetic Resonance Imaging (MRI) of the Brain and Cervical Spine

The MRI scans revealed no structural abnormalities, tumors, or demyelinating lesions that could explain the patient's symptoms.

Electromyography (EMG) and Nerve Conduction Studies (NCS)

EMG and NCS were performed to assess the integrity of the peripheral nerves and muscles in the affected left upper extremity. The results showed normal nerve conduction velocities and no evidence of muscle denervation or myopathy, further supporting the diagnosis of FND.

Somatosensory Evoked Potentials (SSEPs)

SSEPs were recorded to evaluate the sensory pathways from the affected left hand to the brain. The results demonstrated normal cortical responses, indicating intact sensory processing and ruling out any organic sensory abnormalities.

Based on the comprehensive clinical assessment, neurological examination findings, and diagnostic test results, the neurologist confirmed the functional neurological disorder (FND) as the most probable diagnosis in the patient. The neurologist discussed the diagnosis with the patient and provided education about the nature of FND, emphasizing that the symptoms are real but not caused by structural damage to the nervous system. The patient was reassured that FND is a treatable condition and was referred for appropriate multidisciplinary management, including physical therapy, occupational therapy, and cognitive behavioral therapy.

Following the confirmation of the FND diagnosis, the patient was referred for a multidisciplinary treatment approach. This included regular follow-up assessments with the neurologist to monitor the patient's progress and to address any new or persistent symptoms. The patient also engaged in physical therapy and occupational therapy to improve hand function and to learn strategies for managing functional symptoms. Cognitive behavioral therapy was recommended to help the patient cope with the psychological impact of FND and to develop skills for reducing stress and anxiety, which may have contributed to the development of functional symptoms. The patient's progress was regularly assessed through follow-up visits with the multidisciplinary team, and adjustments to the treatment plan were made as needed.

## Discussion

The unexpected comprehensive loss of palmar sensation and motor function across the hand prompted a broad differential diagnosis, encompassing reflex sympathetic dystrophy (RSD), nerve shock or neurapraxia, and conversion and somatoform disorders. The conventional explanations were inadequate in elucidating the observed phenomena due to the acute, transient, extensive sensory and motor loss in multiple sensory territories and muscles, extending beyond the zone of trauma. The innervation of these affected areas is more proximal or upstream compared to the zone of trauma, and given the absence of a high lesion, neurapraxia alone could not account for the observed loss of function. Reflex sympathetic dystrophy (RSD), also known as complex regional pain syndrome type 1, is a chronic pain disorder that typically arises after a minor limb injury [5]. It is characterized by a range of symptoms, including spontaneous disproportionate pain, hyperalgesia, altered skin temperature and color changes, motor function impairment, and vascular abnormalities [6]. The condition is multifactorial, with potential pathophysiological mechanisms including classic and neurogenic inflammation and maladaptive neuroplasticity [5]. However, the symptomatology in our particular case deviated from the aforementioned typical presentation. Such presentations are very rare based on our experience. Aside from motor function impairment, the patient did not manifest any of these characteristic features. Notably, the chronic nature and the profoundly disproportionate pain associated with RSD stand in contrast to the rapid resolution of symptoms observed in this instance.

Similarly, the possibility of nerve shock or neurapraxia seemed implausible. Neuropraxia is recognized as the mildest form of traumatic peripheral nerve injury, characterized by localized, segmental demyelination at the site of the injury. The primary consequence of this type of injury is a blockage in the conduction of nerve signals, leading to transient symptoms such as weakness or sensory disturbances such as paresthesia. Importantly, neuropraxia typically has a favorable prognosis, with spontaneous remyelination and recovery of nerve function expected to occur within a timeframe of about three months, depending on the extent of the injury and the specific nerve involved [7]. However, in our case, the extensive loss of sensation and movement encompassing the entire hand, coupled with an immediate and complete recovery, did not align with the typical clinical course of neurapraxia, which generally entails a more protracted recovery period.

On the other hand, the clinical evidence strongly supports the diagnosis of FND, specifically its subtype, functional movement disorder (FMD), in the case of the patient with transient hand dysfunction. The rapid onset and equally rapid resolution of symptoms, combined with the lack of identifiable structural damage, are hallmark features of FMD. Additionally, the inconsistency in the sensory loss pattern and the normal results from comprehensive neurological and imaging studies further corroborate this diagnosis.

FND denotes "clinical syndromes consisting of symptoms and signs of genuinely experienced alterations in motor, sensory, or cognitive performance that are distressing or impairing and manifest 1) one or more patterns of deficits consistent predominantly with dysfunction of the nervous system and 2) variability in performance within and between tasks" [4].

This diagnosis is informed by a synthesis of the patient's symptoms, the characteristic features of FND and FMD, and comprehensive neurological evaluations and diagnostic tests to rule out other pathologies.

FND, including FMD, is characterized by a wide spectrum of symptoms encompassing both motor and sensory manifestations, with no underlying structural damage. Motor symptoms such as tremors and weakness are prominent in FMD, but sensory symptoms, such as the numbness and loss of aROM displayed by the patient, are also integral to its presentation [4]. The inconsistency and variability of these symptoms, particularly the transient nature and rapid resolution of the numbness, are key indicators of FND. Such fluctuating symptoms, which are atypical in classical neurological disorders, point toward an FMD diagnosis.

Crucially, the patient's sensory loss during the physical examination did not follow typical anatomical patterns. This inconsistency is often observed in FMD and helps distinguish it from other neurological disorders with more predictable sensory deficits. The absence of significant psychological history in the patient does not diminish the likelihood of FND, as psychological stressors are not a prerequisite for symptom onset. Physical factors such as injury or illness can also precipitate FND by causing unexpected sensory experiences [4].

Although the patient did not report any significant psychological history, it is important to consider potential psychological factors that may have contributed to the development of FND. Stressors, whether recognized or unrecognized by the patient, can play a role in the manifestation of FND symptoms. In this particular case, the actual trauma may have acted as a stressor.

In the context of hand surgery, it is crucial to adopt a multidisciplinary approach when managing patients with suspected FND. This may involve collaboration with neurologists, psychiatrists, and occupational therapists to provide comprehensive care and support for the patient. Treatment strategies may include patient education, physical therapy, and cognitive behavioral therapy to address the underlying psychological factors and promote functional recovery [4].

The long-term outcomes and prognosis of FND cases, such as the one presented in this manuscript, can vary depending on the individual patient and the specific subtype of FND. Generally, patients with FMD have a better prognosis compared to those with other subtypes of FND, such as functional seizures or functional sensory deficits [8]. However, the course of FMD can be variable, with some patients experiencing persistent or recurrent symptoms while others achieve complete remission [9]. The factors that may influence the prognosis include early diagnosis, patient acceptance of the diagnosis, and engagement in appropriate multidisciplinary treatment [10]. The long-term follow-up and monitoring of patients with FND are crucial to assess their response to treatment and to identify any potential relapses or new symptoms that may require further intervention.

In this patient's scenario, the lack of internal damage or foreign objects found in surgical and radiological evaluations, combined with the rapid and complete symptom resolution, substantiates the diagnosis of FMD. This case exemplifies the need for considering FND in atypical presentations of hand dysfunction post minor injury.

## Conclusions

This case of a 32-year-old female with transient hand dysfunction after a minor injury exemplifies the diagnostic complexities of FND, particularly FMD, and emphasizes the importance of a thorough neurological assessment in situations where conventional diagnostic approaches do not explain the observed phenomena. It underscores the need for detailed clinical evaluation and, if necessary, neurophysiological testing to accurately diagnose FND and FMD.

FND should be considered in atypical presentations of hand dysfunction, as early recognition and diagnosis are crucial for effective management. This case also highlights the need for further research to better understand the mechanisms underlying FND and to develop evidence-based treatment strategies. Enhanced awareness and multidisciplinary approaches are essential in improving outcomes for patients with FND.

## Figures and Tables

**Figure 1:**
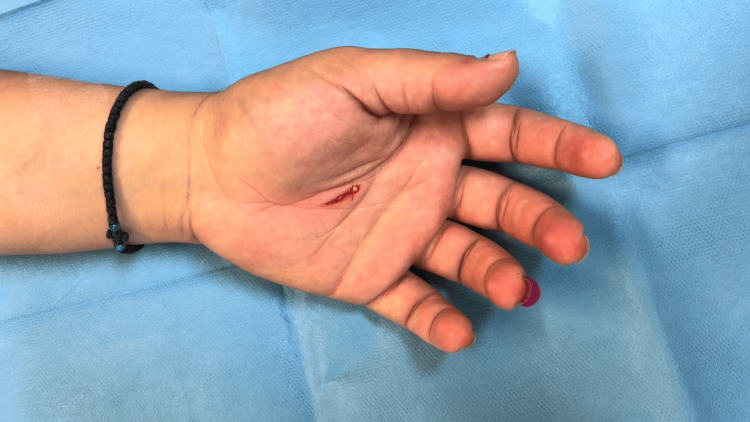
A 1 cm stab wound between the third and fourth metacarpals in the left palm, causing a complete loss of sensation and active range of motion in the entire hand, despite the injury's localized nature.

## Appendices

Figure 1 shows the stab injury between the third and fourth metacarpals.
